# Genetic characterisation of farmed rainbow trout in Norway: intra- and inter-strain variation reveals potential for identification of escapees

**DOI:** 10.1186/1471-2156-9-87

**Published:** 2008-12-16

**Authors:** Kevin A Glover

**Affiliations:** 1 Institute of Marine Research, PO Box 1870, Nordnes, N-5817 Bergen, Norway

## Abstract

**Background:**

The rainbow trout (*Oncorhynchus mykiss*) is one of the most important aquaculture species in the world, and Norway is one of the largest producers. The present study was initiated in response to a request from the Norwegian police authority to identify the farm of origin for 35 escaped rainbow trout captured in a fjord. Eleven samples, each consisting of approximately 47 fish, were collected from the three farms operating in the fjord where the escapees were captured. In order to gain a better general understanding of the genetic structure of rainbow trout strains used in Norwegian aquaculture, seven samples (47 fish per sample) were collected from six farms located outside the region where the escapees were captured. All samples, including the escapees, were genotyped with 12 microsatellite loci.

**Results:**

All samples displayed considerable genetic variability at all loci (mean number of alleles per locus per sample ranged from 5.4–8.6). Variable degrees of genetic differentiation were observed among the samples, with pair-wise *F*_ST _values ranging from 0–0.127. Self-assignment tests conducted among the samples collected from farms outside the fjord where the escapees were observed gave an overall correct assignment of 82.5%, demonstrating potential for genetic identification of escapees. In the "real life" assignment of the 35 captured escapees, all were excluded from two of the samples included as controls in the analysis, and 26 were excluded from the third control sample. In contrast, only 1 of the escapees was excluded from the 11 pooled samples collected on the 3 farms operating in the fjord.

**Conclusion:**

Considerable genetic variation exists within and among rainbow trout strains farmed in Norway. Together with modern statistical methods, this will provide commercial operators with a tool to monitor breeding and fish movements, and management authorities with the ability to identify the source of escapees. The data generated in this study were used by the Norwegian police to initiate an investigation of the company operating the three farms in the fjord where escapees were observed.

## Background

The rainbow trout (*Oncorhynchus mykiss*) is one of the most important cultured fish in the world, and is reared in over 65 countries [1]. In 2006, global production exceeded 550 000 tonnes, and Norway, which is one of major producing countries, accounted for 78 000 tonnes in the same year [2].

In the 1990's, only 1–2% of world aquaculture production was based upon genetically improved stocks [3]. The first successful family based genetic breeding programs for fish were initiated for the rainbow trout and Atlantic salmon (*Salmo salar *L.) in Norway in the early 1970's [4]. Since then, mass selection programs for a number of fish species have been initiated throughout the world (e.g., [5-7]), and the number of breeding programs continues to increase. However, for a number of fish species reared in culture, even major strains are often poorly or only sporadically characterised at the molecular genetic level, and, the use of genetic markers for breeding assistance is not widespread. A number of population genetic studies of wild [8-11], and hatchery [12-14] rainbow trout strains have been conducted in the USA. In contrast, only one major study of European rainbow trout strains has been published [15].

In Norway, rainbow trout production is almost entirely based upon grow-out in marine cages. Some escapement of cage reared fish into the wild is inevitable, and in the period 1998–2007, 8 000–253 000 (83 000 average) annual rainbow trout escapes were reported to the Norwegian Directorate of Fisheries (NDF) [2]. However, these numbers are thought to be an underestimation due to underreporting of escapement [16]. The rainbow trout is not native to Europe, and consequently, escapees cannot cause direct genetic effects on wild populations. Nevertheless, indirect genetic effects (e.g., [17],) and ecological effects on native salmonid populations (Atlantic salmon and brown trout *Salmo trutta *L.), may occur. Consequently, better control and monitoring of escapees is required. In a recent study, Glover et al. [18] reported, for the first time, successful identification of farmed escaped Atlantic salmon to both farm and cage of origin. This was achieved by comparing individual genetic profiles of escapees with group genetic profiles for 16 samples of Atlantic salmon taken from seven farms. That study built upon the fact that clear genetic differences have been reported among the major Atlantic salmon strains farmed in Norway [19].

The present study was initiated in response to a request by the NDF and the Norwegian police. These authorities requested genetic analyses of rainbow trout that had been captured in a fjord, in order to assist in the identification of their most likely farm(s) of origin. At the time of investigation, however, the genetic structure within and among rainbow trout strains used by the Norwegian aquaculture industry was not known. Consequently, the present study aimed to 1) investigate the extent of genetic variation within and among rainbow trout strains used in Norwegian aquaculture, 2) study the potential of genetic assignment to identify rainbow trout to strain and farm of origin, and 3) to attempt to identify the farm(s) of origin for a group of escaped rainbow trout in a Norwegian fjord based upon a "real-life" escapement episode.

## Methods

### Samples

For legal reasons, the names and locations of all farms and samples used in this study, including exact dates of collection, are anonymous. The present study was initiated after the NDF received several reports detailing observations of escaped rainbow trout in a Norwegian fjord in 2008. No farm in the area reported any losses of fish. A total of three operational rainbow trout farms, all owned by the same company, were located in the inner part of the fjord. All were sited within a 5 km radius from where the escapees were captured, and the closest alternative source for the rainbow trout escapees was >130 km distant by sea. The escapees were uniform in size (1.5–3.8 kg), and overlapped with the size range of rainbow trout held on the three farms in the vicinity. Losses of cage-reared salmonids are often detected in the local area by a sudden increase in the catch per unit effort [20]. Whilst the circumstances surrounding the escapement indicated that the most likely source of escapement was from one or more of these three farms, the Norwegian police required additional evidence to initiate a formal investigation. A simple question was posed. Does the genetic profile of the escapees fit in with one or more of the suspected farms, or can they be excluded as potential sources of the escapement? In order to address this question, a total of 11 samples, representing all cages on these farms, were taken (Table 1). Multiple cage sampling was conducted as the fish reared on the three farms were declared as a non-random and unidentified mixture of two genetic strains, and it was essential to sample all possible sources of the escapees. These farms (farms 7–9, samples 8–18) are here on referred to as the suspected farms/samples collectively. In addition, the NDF collected 35 escaped rainbow trout by contacting local fishermen who had captured escapees in the area in the period January-March 2008.

**Table 1:** Samples used in the present study.

Sample	Farm or Location	Sample size (n)	Specification of sample	Genetic strain
1	1	47	Slaughterhouse A. (mixed cages)	Not specified
2	2	47	Slaughterhouse A. (mixed cages)	SSF
3	3	47	Slaughterhouse B. (mixed cages)	ISV
4	4	47	Single production cage sampled (sea)	AG
5	5	47	Single tank sampled (freshwater)	AL
6	5	47	Single tank sampled (freshwater)	SB
7	6	47	Single production cage sampled (sea)	AG
8	7	47	Single production cage sampled (sea)	AG + ISV mixed
9	7	47	Single production cage sampled (sea)	AG + ISV mixed
10	7	47	Single production cage sampled (sea)	AG + ISV mixed
11	7	47	Single production cage sampled (sea)	AG + ISV mixed
12	8	47	Single production cage sampled (sea)	AG + ISV mixed
13	8	47	Single production cage sampled (sea)	AG + ISV mixed
14	8	47	Single production cage sampled (sea)	AG + ISV mixed
15	9	47	Single production cage sampled (sea)	AG + ISV mixed
16	9	47	Single production cage sampled (sea)	AG + ISV mixed
17	9	47	Single production cage sampled (sea)	AG + ISV mixed
18	9	47	Single production cage sampled (sea)	AG + ISV mixed
19	10	35	Escapees captured in sea close to farms 7–9	Unknown

Samples 8–18 were collected on the three rainbow trout farms located in the fjord where the escapees were captured. Samples 1–7 were collected from different locations throughout Norway and were not regarded as potential sources of origin for the escapees (sample 19). SSF = Stolt Sea Farms, ISV = Ilsvåg, AG = Aqua Gen, AL = Alf Lone, SB = Salmobreed.

To investigate genetic variation observed within and among rainbow trout strains used in Norwegian aquaculture in general, seven samples from other sites were also analysed (Table 1). The locations of these samples were such that they were not regarded as potential sources for the 35 escapees. Approximately 47 fin clips from individual fish were taken for each sample, and stored in ethanol until DNA extraction.

### Microsatellite screening

DNA was extracted from fin clips using a Qiagen DNAeasy 96 tissue kit. Twelve rainbow trout derived microsatellite loci (Table 2) were amplified in two separate multiplexes using a mixture of the reaction and amplification conditions presented in two earlier articles [12,21] as the start point for optimisation. PCR amplification was performed as a multiplex in 96 well plates with a total reaction volume of 12 μl. For both multiplexes, each reaction consisted of 2 μl DNA, 2.0 mM MgCl_2_, 0.2 mM dNTP's and 0.5 U Go *Taq *polymerase and the manufacturers' 1× reaction buffer. In all cases, forward primers were labelled with fluorescent dyes for fragment detection. Primer concentrations for both multiplexes are presented (Table 2). Amplification was performed in an Eppendorf Master Cycler using the following programme for multiplex 1: 95°C for 10 minutes, then 2 cycles of 94°C for 1 minute, 62°C for 45 seconds and 72°C for 2 minutes, then 29 cycles of 94°C for 1 minute, 58°C for 45 seconds and 72°C for 2 minutes, and finishing with a 72°C extension for 45 minutes and hold at 4°C, and for multiplex 2: 95°C for 10 minutes, 29 cycles of 94°C for 1 minute, 58°C for 45 seconds and 72°C for 2 minutes, and finishing with a 72°C extension for 45 minutes and hold at 4°C. PCR fragments were separated using an ABI 3730 sequencing machine and sized relative to the Applied Biosystem GeneScan™-500 LIZ™ size standard. Alleles were scored using automatic binning implemented in the Genmapper software (V4.0) with manual checking before data were exported for further analyses.

**Table 2:** Summary data for 12 microsatellite markers.

Hexaplex 1				Hexaplex 2			
Marker	Primer μM	ABI dye	Allele size	Marker	Primer μM	ABI dye	Allele size
*OMM5132*^1^	0.25	6-Fam	94–126*	*OMM5177*^1^	0.1	6-Fam	115–142
*OMM5047*^2^	0.1	6-Fam	259–278*	*OMM1051*^4^	0.075	6-Fam	210–290
*OMM1303*^3^	0.2	Pet	286–362	*OMM5264*^1^	0.12	Pet	104–117
*OMM5007*^2^	0.08	Ned	155–199	*OMM1097*^5^	0.1	Ned	213–319*
*OMM5233*^1^	0.1	Vik	112–138	*OMM1088*^5^	0.075	Vik	113–159
*OMM1008*^4^	0.1	Vik	258–279	*OMM1325*^3^	0.075	Vik	278–296*

Concentration is for each primer (i.e., forward and reverse). ^1 ^= [36], ^2 ^= [37], ^3 ^= [38], ^4 ^= [39], ^5 ^= [40]. * denotes marker displaying one or more alleles separated by 1 bp.

Following the main screening, DNA from the 35 escapee fish was isolated for a second time. These samples, in addition to 60 DNA samples randomly selected from the first screening were then re-analysed (PCR amplification followed by allele detection in ABI) in order to check for accuracy of genotyping. Routinely carrying out such checks in DNA data sets has recently been recommended as standard practice [22,23].

### Statistical analysis

Genepop V3.4 [24] was used to test for deviation from Hardy Weinberg equilibrium, calculate pair-wise *F*_ST _values among the samples, and test for sample differentiation using the Fishers method (Markov chain parameters: dememorization number 1000, number of batches 100, iterations per batch 1000). FSTAT V2.9.3.2 [25] was used to compute allele frequencies for all loci in all samples, and estimate allele richness. TFPGA V1.3 [26] was used to calculate pair-wise genetic distance according to Nei's 1978 genetic distance [27], calculate heterozygosity for all samples across all loci, and produce the UPMGA diagram. Assignment tests were conducted using Geneclass V1.0.02 [28]. Self-assignment was conducted using the direct assignment option, Bayesian method of computation, and the leave one out sub-option. The 35 escapees were assigned to farm samples using a mixture of direct assignment, which places each individual into the sample that it is most similar to irrespective of absolute value of genetic similarity, in addition to probability based exclusion, which rejects individuals from samples at a set probability, both using the Bayesian method of computation.

In order to investigate different escapement and identification scenarios, self-assignment simulations were conducted on several data sub-sets. First, self-assignment was conducted using only the samples collected from outside of the fjord where the escapees were captured (samples 1–7). Second, self-assignment was conducted for samples 1–8, 10, 12 and 15. This sub-data set included all samples collected from outside the fjord where the escapees were captured, in addition to a sample from each of the three farms within the fjord. For farm 7 however, two samples were represented. This is because two genetically distinct groups of fish were observed on this farm (see results). Third, self-assignment was conducted among all farm samples (1–18).

In order to identify the source of origin for the 35 escapees, a combination of direct assignment, in addition to exclusion (at 0.05 probability), were carried out using a genetic baseline consisting of all samples collected from the three farms located in the vicinity of the escapees (samples 8–18), and three samples collected from other locations (samples 5–7). Due to the high degree of miss-assignment observed for the self-assignment simulations within the entire data set (see results), and, that none of the samples collected from outside of the fjord were considered to be real alternative sources of origin, only three of these samples, randomly selected to serve as controls, were included for these computations.

## Results

### Genotyping quality

The control re-screening of 95 DNA samples produced identical genotype scores for ten of the markers. For *OMM5233 *however, 7 of the 95 individuals produced genotyping discrepancies between first and second runs. This occurred as the shorter allele in many individuals was significantly lower in amplitude than the longer allele, and was as a consequence inconsistently scored. For *OMM5132*, a single genotyping error was observed. This individual was incorrectly genotyped as a homozygote in the first run. Consequently, with the exception of locus *OMM5233*, genotyping was highly consistent. This is despite the fact that four of the loci (*OMM1097*, *OMM1325*, *OMM5047*, *OMM5132*) displayed one or more alleles just a single base pair distant from a neighbouring allele.

Potential departure from Hardy Weinberg equilibrium was examined in all loci and samples. A total of 40 significant departures out of 228 tests were observed (*P *< 0.05). Looking at loci first, with the exception of *OMM5233 *(6 departures), and *OMM1051 *(14 departures), departures were evenly distributed among loci (1–3 departures per locus). A similar pattern of distribution of departure from Hardy Weinberg equilibrium was observed for samples across loci. Most samples displayed 0–3 significant departures with the exception of sample 2 (5 departures), sample 14 (5 departures), and sample 15 (4 departures). Due to genotyping inconsistency and/or extensive departure from Hardy Weinberg equilibrium, both *OMM5233 *and *OMM1051 *were excluded from all further statistical analyses. Following this, and implementation of Bonferroni correction for multiple independent tests (new *P *= 0.005), only two departures, both in sample 14, remained significant.

### Genetic variation within samples

The amount of genetic variation observed in each sample for each locus is presented (Table 3). A total of 136 alleles were observed in the entire data set (mean per locus = 11.3), ranging from 5–24 for loci across samples, and 65–103 for samples across loci. For the samples taken outside of the fjord where the escapees were captured (samples 1–7), total number of alleles per sample ranged from 65–93, whereas the total number of alleles for the samples collected on farms located in the fjord where escapees were captured (samples 8–18) ranged from 83–103. Individual samples contained 48–76% of the allelic variation observed in the pooled material. Observed heterozygosity averaged across all 12 loci ranged from 0.65–0.79 among all of the samples (Table 3).

**Table 3:** Allelic variation observed at 12 loci in 19 samples of rainbow trout.

Sample	N	Locus (all have *OMM *as pre-fix)												Allele summary			
		
		*1008*	*1051*	*1088*	*1097*	*1303*	*1325*	*5007*	*5047*	*5132*	*5177*	*5233*	*5264*	A_T_	A_M_	A_A_	Ho
1	47	4	6	7	8	6	4	6	5	6	4	8	4	68	5.7	66.2	0.67
2	46	6	8	7	10	9	7	8	7	9	5	8	5	89	7.4	84.9	0.74
3	39	4	6	7	9	5	4	7	3	7	4	6	3	65	5.4	63.7	0.68
4	47	5	11	8	13	8	6	7	6	12	4	8	5	93	7.8	90.2	0.77
5	44	6	7	6	9	7	4	7	4	9	4	10	5	78	6.5	72.9	0.71
6	46	5	9	6	9	8	5	10	4	9	4	4	5	78	6.5	75.3	0.73
7	46	4	5	7	10	6	5	7	3	7	4	7	3	68	5.7	65.3	0.65
8	46	5	8	7	12	9	3	8	7	7	4	9	4	83	6.9	77.3	0.70
9	42	5	9	7	13	8	5	7	6	8	4	9	4	85	7.1	79.3	0.70
10	43	6	13	8	11	11	6	10	6	12	5	7	5	100	8.3	93.8	0.77
11	46	6	12	9	10	13	5	8	7	12	5	7	5	99	8.3	93.1	0.78
12	41	6	10	8	14	11	5	11	7	11	5	7	5	100	8.3	94.8	0.74
13	30	6	11	8	11	11	6	8	7	9	4	8	4	93	7.8	92.4	0.77
14	46	6	10	8	14	11	5	9	7	10	5	10	4	99	8.3	92.7	0.77
15	45	6	12	8	14	11	6	11	7	9	5	8	5	102	8.5	94.0	0.77
16	45	6	11	8	15	11	6	11	7	11	5	7	5	103	8.6	95.9	0.77
17	43	6	11	8	11	10	6	10	7	10	5	9	5	98	8.2	92.8	0.77
18	43	6	10	8	15	11	6	11	8	11	5	8	4	103	8.6	97.2	0.76
19	35	6	11	8	13	12	5	9	6	11	4	9	5	99	8.3	96.8	0.79

Total	820	6	16	9	24	14	7	14	8	17	5	11	5	136	11.3	100.0	---

*N *= mean number of observations per locus, A_T _= total number of alleles, A_M _= mean number of alleles, A_A _= allelic richness based upon a minimum sample size of 28 diploid individuals, Ho = observed heterozygosity.

### Genetic variation among samples

**Figure 1:**
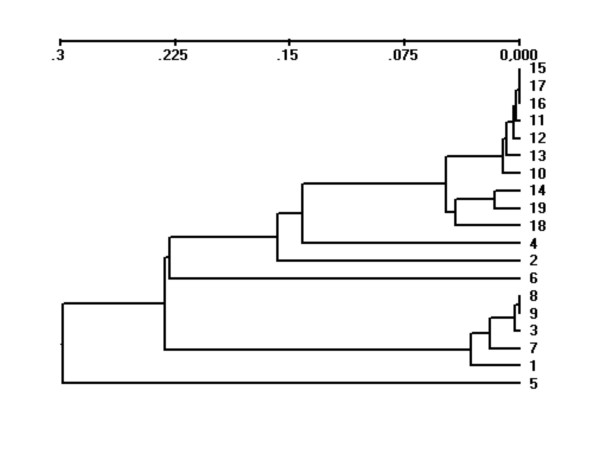
**UPMGA diagram based upon Nei's (1978) unbiased genetic distance among 19 samples of rainbow trout**.

A matrix of genetic distances among all samples as estimated by *F*_ST _and Nei's 1978 genetic distance [27] is presented (additional file 1). Pair-wise *F*_ST _values ranged from 0–0.127, and in total, 21 of the 171 pair-wise comparisons were greater than 0.1. The greatest differentiation was observed between samples 5 and 7, and, the majority of the highest values (for example those over 0.1) involved samples 1, 5, 7. Genetic relationships among the samples are also presented using Nei's 1978 [27] genetic distance (Fig. 1). The genetic material reared in the three farms under suspicion (samples 8–18), grouped into two major clusters. One of the clusters was genetically distinct from all of the other samples in the total data set, and involved samples 10–18. Within this cluster, all pair-wise *F*_ST _values were under 0.022, and the majority were smaller than 0.01. In addition, the vast majority of the few pair-wise sample comparisons that were not genetically differentiated according to the Fishers method involved samples from this cluster (additional file 1). The second major cluster involving the samples originating from the three farms under suspicion involved samples 8 and 9. This cluster also included control samples 1, 3 and 7.

Pair-wise *F*_ST _values between samples 8 and 9 were very small, and did not display significant differentiation (additional file 1). Likewise, pair-wise *F*_ST _values between samples 10 and 11 were very small, and lacked significance (additional file 1). These pairs of samples were both reported to have been the result of splitting fish from two to four cages prior to the NDF taking samples of the fish.

### Self-assignment simulations

For the computation including samples 1–7, overall correct self-assignment was calculated at 83%, and ranged from 63–96% for individual samples (Table 4). When self-assignment was computed for samples 1–7, 8, 10, 12 and 15, overall correct self-assignment dropped to 61%, and, when computed for the entire data set (escapees excluded), correct overall self-assignment dropped further to 38%.

**Table 4:** Results of self-assignment tests.

Sub-set 1												
Sample	1	2	3	4	5	6	7	Correctly assigned				

1	**32**	-	12	-	-	-	3	68%				
2	-	**43**	-	2	-	-	1	93%				
3	3	1	**28**	-	-	-	7	72%				
4	-	2	-	**45**	-	-	-	96%				
5	1	-	-	-	**40**	3	-	91%				
6	-	1	-	2	-	**43**	-	94%				
7	11	-	6	-	-	-	**29**	63%				
Overall								83%				

												
Sub-set 2												

Sample	1	2	3	4	5	6	7	8	10	12	15	Correctly assigned

8	3	-	15	-	-	-	8	**18**	-	-	1	40%
10	-	2	-	1	-	1	-	**-**	**14**	17	8	36%
12	-	1	-	3	-	2	-	-	10	**18**	8	43%
15	-	3	-	1	1	-	-	-	12	14	**14**	31%
Overall												37%

Sub-set 1 represents a full matrix of self-assignment among samples 1–7. Sub-set 2 represents a partial matrix of self-assignment for samples 8, 10, 12 and 15 combined in sub-set 1. Numbers in bold represent exact numbers of fish correctly assigned to their sample of origin.

Miss-assignment was not random among the samples. Taking sub-set 1 first (Table 4), samples 1, 3 and 7 displayed a moderate degree of miss-assignment among themselves. This is consistent with the fact that samples 1, 3 and 7 represent part of a cluster which also includes samples 8 and 9 (Fig. 1). The relationship between pair-wise genetic distance and miss-assignment is further apparent in the partial matrix for sub-set 2 (Table 4), where sample 8 displayed a high degree of miss-assignment to samples 1, 3 and 7. However, in contrast to samples 10, 12 and 15, sample 8 did not display any significant degree of miss-assignment to the other cage sampled on the same farm (sample 10) nor cages sampled in neighbouring farms (samples 12 and 15) originating from the same material that was reported to be mixed.

### Assignment of the escapees

Assignment of the 35 rainbow trout escapees is presented (Table 5). Direct assignment to sample resulted in the 35 escapees being scattered evenly among the samples used to generate the baseline. This is consistent with the patterns of miss-assignment described above. Nevertheless, when data from the farms were combined (Table 5), 32 of the 35 escapees were directly placed into the samples collected from the farms located in the fjord where the escapees were captured. Direct assignment places each individual into the sample that it is genetically most similar to, irrespective of the degree of similarity. In the context of forensics, the most robust form of genetic identification is through exclusion rather than inclusion. Taking the samples collected from outside the fjord where the escapees were captured first, at a significance level of *P *= 0.05, all of the 35 escapees were excluded from samples 5 and 6, and, 26 of the 35 escapees were excluded from sample 7. For nine of the 11 samples collected on the three farms under suspicion, 19–23 of the 35 rainbow trout could not be excluded on an individual sample basis. However, for samples 8 and 9, only 10 and 11 of the escapees could originate from those samples at the calculated level of exclusion. Interestingly, samples 8 and 9 are those that display the greatest degree of genetic similarity to sample 7 (additional file 1, Fig. 1), where a similar level of exclusion was estimated. When samples from the three farms under suspicion were combined, only one out of 35 escapees could be excluded.

**Table 5:** Assignment of 35 captured rainbow trout escapees.

Single samples													
8	9	10	11	12	13	14	15	16	17	18	5	6	7

Direct assignment													
6	4	2	1	3	2	1	3	5	2	3	0	1	2
Not excluded at 0.05													
10	11	19	20	20	19	21	20	22	20	23	0	0	9
Pooled samples													

Farm 7 (samples 8–11)			Farm 8 (samples 12–14)			Farm 9 (samples 15–18)			Suspected farms (samples 8–18)			Control farms (samples 5–7)	

Direct assignment													
13			6			13			32			3	
Not excluded at 0.05													
31			31			28			34			9	

The genetic baseline comprises of 11 samples taken from the three farms in the fjord where the escapees were captured, and 3 control samples taken from two farms outside the region.

## Discussion

Results of the "real-life" assignment for 35 escaped rainbow trout did not identify a single farm, or cage of origin. This was expected, since the three farms operating within the fjord where the escapees were captured, shared genetic material. This was confirmed by the low pair-wise *F*_ST _values among many of these samples, and the resultant miss-assignment among them when simulating with the data (Table 4). Nevertheless, all 35 escapees were excluded from two of the samples included as controls in the baseline for this analysis, and, the majority were excluded from the third control sample. As only one of the 35 escapees could be excluded from having originated from one or more of the three farms located in the fjord where the escapees were captured, and the nearest other rainbow trout farm was >130 km distance by sea, the genetic analyses presented here provide clear evidence that the rainbow trout escapees probably originated from one or several of the three farms under suspicion. As a single company owned all three farms, this permitted the Norwegian police to initiate an investigation of the company.

Significant among-sample variation is a pre-requisite for genetic assignment, with greater differentiation increasing accuracy [28-30]. The *F*_ST _values observed among samples in the present study are similar to those observed among some of the major Atlantic salmon breeding strains used in aquaculture [19], and, similar to those observed among cage reared salmon [18]. Furthermore, whilst the overall correct self-assignment reported here (83%) among the 7 samples collected outside the location where escapees were captured, is slightly less than reported for 12 European rainbow trout strains (92%) [15], it is greater than observed by Glover et al. [18] in a study of cage-reared Atlantic salmon. Comparison to the latter study is important, because it described, for the first time, use of genetic assignment to identify the cage and farm of origin for a group of escaped farmed Atlantic salmon.

Throughout the world, over 75 strains of rainbow trout have been produced in culture [31], and over 65 countries have reported aquaculture activity of this species [1]. Norway is one of the largest producers, accounting for approximately 15% of global production in 2006 [2]. In 2008, when samples for this study were collected, the majority of the Norwegian production was based upon five strains (Aqua Gen AS, Salmobreed AS, Ilsvåg, Alf Lone and Stolt sea farms). Data from this study demonstrated variable but nevertheless significant genetic differentiation among samples of fish reported to originate from these strains, with clear opportunities for genetic assignment.

The degree of genetic differentiation observed among the samples in this study (highest pair-wise *F*_ST _= 0.127) is within the range of values observed in other studies of rainbow trout throughout the world. Among 12 European strains, Gross et al. [15] reported an average *F*_ST _of 0.14. When looking specifically among Danish and Finnish strains however, these authors reported a mean *F*_ST _of 0.1 and 0.05 respectively. Among 24 anadromous samples in California, Aguilar & Garza [11] reported the largest pair-wise *F*_ST _value of 0.171, whilst Deiner et al. [32] reported pair-wise *F*_ST _values as high as 0.385 among 20 land-locked populations in California. Johnson et al. [12] reported an *F*_ST _of 0.0481 between two hatchery strains of rainbow trout in West Virginia, whilst Silverstein et al. [14] reported an average *F*_ST _of 0.089 among three distinct hatchery strains in USA.

It is difficult to make direct comparisons of genetic variation to other studies where different marker sets have been used. However, a tentative comparison with the 12 European strains analysed by Gross et al. [15], analysed by 10 microsatellite loci (no marker overlap with present study) indicate a similar range of mean number of alleles per sample between studies (3.2–7.2 for 12 European strains, 5.4–7.8 for samples 1–7 in this study). In a study of three domesticated rainbow trout strains in USA, Silverstein et al. [14] reported mean number of alleles per locus per sample of 8.1–9.6, with 9 microsatellite loci. A direct comparison with the study of two hatchery strains maintained at the National Centre for Cool and Cold Water Aquaculture in USA (NCCCWA) [12] is possible as nine of the loci analysed by these authors overlapped with loci used here. These authors reported an average number of alleles per strain from 11.1–13.6, which is much higher than the values reported here. This was also clear on an individual locus basis. For example, 15–22 alleles per strain for locus *OMM1097 *was reported in the strains reared in the NCCCWA, whilst only 8–13 alleles were observed per sample for samples 1–7 in the present study. Whilst 15 alleles were observed for *OMM1097 *in samples 16 and 18, these were reported to represent a mixture of strains. Consequently, although allelic variation is positively linked with sample size [33,34], and the sample sizes screened by Johnson et al. [12] ranged from 60–67, in contrast to 39–47 in the present study, it cannot be excluded that rainbow trout strains used in Norwegian aquaculture display reduced allelic diversity compared to the NCCCWA strains. This is consistent with the manner by which rainbow trout have been distributed beyond their native range [31,35], and the potential for multiple founder effects and/or bottlenecks. However, it is also possible that the observed differences may be a consequence of sampling. Samples taken at the NCCCWA were obtained directly from the breeding stock, whilst in the present study, samples were taken from production units, which may not necessarily contain all of the genetic variation present in the breeding nucleus. This requires further elucidation.

In the present study, two samples (4 and 7) were reported to originate from the Aqua Gen strain. Both pair-wise *F*_ST _values and total number of alleles varied greatly between the two samples. Whilst it cannot be excluded that this is a result of incorrect information regarding the origin of these samples, or mixing of genetic material from several sources, it is possible that this is a consequence of two different smolt-producers delivered rainbow trout to the two marine farms where samples 4 and 7 were collected. Genetic differences between samples originating from the same strain can be generated by non-random sorting of gametes, fertilised eggs, fry, and smolts in the process of distributing genetic material from the central breeding stations, to marine producers, as has been previously suggested [18].

The detailed examination of genetic variation in and among all cages of rainbow trout collected from the three farms under suspicion, highlight the possibility to use genetic tools to monitor production. For example, this could assist in the reconstruction and verification of fish movements among cages and sites, check for illicit (re-)production of a specific strain by a competing company or customer, or confirm genetic background to a new delivery of fish. This potential is best illustrated by looking at the four samples taken on farm 7 (samples 8–11). Samples 8 and 9 were collected from two adjacent cages, and were reported to have been recently split from a single cage prior to sampling. This was also reported for the pair of samples 10 and 11. The very low *F*_ST _values observed between samples 8 and 9, and, 10 and 11, combined with the almost identical total number of alleles observed for each pair of samples (83 and 85, 100 and 99, for samples 8, 9, 10 and 11 respectively), are consistent with the information supplied by the farmer. However, the very clear genetic differences between pairs of samples 8 and 9 vs. 10 and 11, as revealed by *F*_ST _and allelic variation, provided extra information. Clearly, these two groups of fish did not represent a homogenous mixture of fish from a single source.

## Conclusion

This study represents the first molecular genetic characterisation of farmed rainbow trout in Norway, one of the worlds largest producers. A significant amount of genetic variation was observed both within, and, among the samples examined. Among sample variation reflected genetic differences between groups of fish reared on different farms, in different cages on the same farm, and among strains. In the "real-life" assignment of 35 rainbow trout escapees, with the exception of one individual, none could be excluded from originating in one or several of the three farms operating in the fjord where they were captured. In contrast, two of the samples used as a control were excluded as potential donors for all of the escapees, and the third sample used as a control was excluded for the majority of the escapees. These data were used by the Norwegian police to initiate an investigation of the company operating the three farms in the fjord. This study, has clearly demonstrated potential for genetic assignment of rainbow trout in aquaculture, providing farmers with the ability to monitor various aspects of production, and management authorities with a tool with which to trace escapees.

## Authors' contributions

KAG designed the study, supervised laboratory work, carried out data analysis, and wrote the article.

## Supplementary Material

Additional file 1 Pair-wise genetic distances among 19 samples of rainbow trout. This matrix of values includes estimations by *F*_ST _in the lower diagonal and Nei's (1978) unbiased genetic distance in the upper diagonal. Click here for file
