# Supplementary material for: Genetic characterisation of farmed rainbow trout in Norway: intra- and inter-strain variation reveals potential for identification of escapees
Source: BMC Genet. 2008 Dec 16;9:87. doi: 10.1186/1471-2156-9-87 (PMC2640418; doi:10.1186/1471-2156-9-87)
Supplement: Additional file 1 — Pair-wise genetic distances among 19 samples of rainbow trout. This matrix of values includes estimations by FST in the lower diagonal and Nei's (1978) unbiased genetic distance in the upper diagonal. [file 1471-2156-9-87-S1.doc]

**Additional file – 1.** **Pair-wise genetic distances among 19 samples of rainbow trout.** Distances are estimated by *F*_ST_ (lower diagonal), and Nei`s 1978 unbiased genetic distance (upper diagonal). * denotes pairs of samples not displaying significant genetic differentiation as estimated by Fishers method and post Bonferroni correction (adjusted *P* =0.0026).

|  | Sample | | | | | | | | | | | | | | | | | | |
| --- | --- | --- | --- | --- | --- | --- | --- | --- | --- | --- | --- | --- | --- | --- | --- | --- | --- | --- | --- |
| Sample | 1 | 2 | 3 | 4 | 5 | 6 | 7 | 8 | 9 | 10 | 11 | 12 | 13 | 14 | 15 | 16 | 17 | 18 | 19 |
| 1 |  | 0.169 | 0.035 | 0.363 | 0.307 | 0.242 | 0.026 | 0.036 | 0.031 | 0.328 | 0.354 | 0.316 | 0.248 | 0.110 | 0.274 | 0.316 | 0.318 | 0.233 | 0.140 |
| 2 | 0.067 |  | 0.183 | 0.183 | 0.190 | 0.164 | 0.197 | 0.156 | 0.147 | 0.184 | 0.174 | 0.206 | 0.157 | 0.118 | 0.151 | 0.170 | 0.181 | 0.140 | 0.078 |
| 3 | 0.018 | 0.067 |  | 0.345 | 0.352 | 0.275 | 0.027 | 0.001 | 0.005 | 0.299 | 0.309 | 0.288 | 0.221 | 0.081 | 0.272 | 0.327 | 0.300 | 0.206 | 0.121 |
| 4 | 0.117 | 0.052 | 0.107 |  | 0.413 | 0.332 | 0.339 | 0.304 | 0.247 | 0.174 | 0.124 | 0.160 | 0.148 | 0.186 | 0.127 | 0.137 | 0.131 | 0.128 | 0.100 |
| 5 | 0.113 | 0.063 | 0.120 | 0.111 |  | 0.232 | 0.343 | 0.327 | 0.307 | 0.324 | 0.310 | 0.325 | 0.298 | 0.253 | 0.267 | 0.289 | 0.277 | 0.335 | 0.226 |
| 6 | 0.089 | 0.051 | 0.094 | 0.087 | 0.075 |  | 0.288 | 0.239 | 0.258 | 0.269 | 0.239 | 0.261 | 0.226 | 0.204 | 0.196 | 0.214 | 0.233 | 0.259 | 0.147 |
| 7 | 0.014 | 0.079 | 0.015 | 0.116 | 0.127 | 0.106 |  | 0.010 | 0.020 | 0.308 | 0.345 | 0.289 | 0.238 | 0.097 | 0.282 | 0.321 | 0.301 | 0.218 | 0.134 |
| 8 | 0.017 | 0.056 | 0.001* | 0.093 | 0.110 | 0.080 | 0.006 |  | -0.004 | 0.266 | 0.271 | 0.242 | 0.185 | 0.062 | 0.225 | 0.275 | 0.250 | 0.166 | 0.092 |
| 9 | 0.015 | 0.051 | 0.003* | 0.077 | 0.102 | 0.084 | 0.012 | -0.002* |  | 0.224 | 0.229 | 0.209 | 0.154 | 0.046 | 0.188 | 0.233 | 0.199 | 0.129 | 0.070 |
| 10 | 0.108 | 0.051 | 0.096 | 0.046 | 0.091 | 0.073 | 0.107 | 0.083 | 0.071 |  | 0.007 | 0.010 | 0.009 | 0.050 | 0.024 | 0.008 | 0.008 | 0.061 | 0.058 |
| 11 | 0.112 | 0.049 | 0.097 | 0.034 | 0.088 | 0.066 | 0.115 | 0.084 | 0.072 | 0.002* |  | 0.007 | 0.009 | 0.064 | 0.003 | 0.003 | -0.006 | 0.053 | 0.043 |
| 12 | 0.110 | 0.062 | 0.099 | 0.048 | 0.098 | 0.076 | 0.107 | 0.082 | 0.071 | 0.004* | 0.003* |  | 0.015 | 0.057 | 0.007 | 0.001 | -0.003 | 0.069 | 0.042 |
| 13 | 0.090 | 0.045 | 0.078 | 0.040 | 0.087 | 0.063 | 0.091 | 0.064 | 0.053 | 0.002* | 0.003* | 0.006* |  | 0.021 | -0.005 | 0.013 | 0.003 | 0.041 | 0.029 |
| 14 | 0.047 | 0.035 | 0.034 | 0.051 | 0.077 | 0.059 | 0.046 | 0.025 | 0.019 | 0.015* | 0.019 | 0.019 | 0.006 |  | 0.053 | 0.070 | 0.061 | 0.044 | 0.016 |
| 15 | 0.095 | 0.044 | 0.090 | 0.035 | 0.079 | 0.056 | 0.101 | 0.074 | 0.062 | 0.007* | 0.001* | 0.003* | -0.002* | 0.016 |  | -0.004 | -0.012 | 0.050 | 0.022 |
| 16 | 0.106 | 0.049 | 0.103 | 0.039 | 0.085 | 0.061 | 0.112 | 0.087 | 0.074 | 0.003* | 0.001* | 0.001* | 0.004* | 0.021 | -0.001* |  | 0.002 | 0.052 | 0.031 |
| 17 | 0.106 | 0.051 | 0.096 | 0.036 | 0.082 | 0.065 | 0.106 | 0.080 | 0.065 | 0.002* | -0.002* | 0.000* | 0.001* | 0.018 | -0.004* | 0.001* |  | 0.059 | 0.028 |
| 18 | 0.085 | 0.042 | 0.074 | 0.037 | 0.097 | 0.073 | 0.085 | 0.058 | 0.046 | 0.018 | 0.016 | 0.022 | 0.012 | 0.013 | 0.015 | 0.016 | 0.018 |  | 0.040 |
| 19 | 0.059 | 0.024 | 0.049 | 0.027 | 0.068 | 0.043 | 0.061 | 0.037 | 0.029 | 0.016 | 0.012 | 0.015 | 0.008 | 0.005* | 0.007 | 0.010* | 0.008 | 0.012 |  |
